# Supplementary material for: Biocompatible Materials Based on Plasticized Poly(lactic acid), Chitosan and Rosemary Ethanolic Extract I. Effect of Chitosan on the Properties of Plasticized Poly(lactic acid) Materials
Source: Polymers (Basel). 2019 May 30;11(6):941. doi: 10.3390/polym11060941 (PMC6631666; doi:10.3390/polym11060941)
Supplement: Supplementary file 1 [file polymers-11-00941-s001.pdf]

Supplementary materials

Table 10. The influence of biocomposites administration on the AST, ALT and LDH activity. Values were presented as mean ± S.D. for 6 animals in a group.

Groups		AST (U/mL)	ALT (U/mL)	LDH (U/mL)
**Control**	**24 hrs**	41.7±2.72	95.3±4.14	342.29±44.55
	**7 days**	42.5±3.07	96.5±3.89	344.33±41.37
**PLA/PEG**	**24 hrs**	43.7±3.37	98.5±5.46	345.72±44.37
	**7 days**	44.8±3.89	98.8±4.33	349.15±40.46
**PLA/PEG/3CS**	**24 hrs**	41.8±2.89	95.5±5.14	344.68±41.33
	**7 days**	42.5±3.37	96.4±5.55	346.22±43.72
**PLA/PEG/6CS**	**24 hrs**	41.3±2.64	96.1±5.46	344.75±42.55
	**7 days**	43.8±3.33	96.7±5.25	347.67±44.37
**PLA/PEG/0.5R**	**24 hrs**	43.5±3.14	97.2±3.89	345.45±39.89
	**7 days**	44.6±3.64	98.6±5.72	347.83±42.27
**PLA/PEG/3CS/0.5R**	**24 hrs**	42.4±3.46	97.2±5.89	345.29±42.64
	**7 days**	43.9±3.14	97.9±6.05	347.43±40.89
**PLA/PEG/6CS/0.5R**	**24 hrs**	42.7±3.72	97.4±5.33	345.52±44.55
	**7 days**	43.9±3.55	98.7±6.14	348.83±43.46

Table 11. The influence of biocomposites administration on the serum urea and creatinine concentration. Values were presented as mean ± S.D. for 6 rats in a group.

**Groups**		**urea (mg/dL)**	**creatinine (mg/dL)**
**Control**	**24 hrs**	37.2±3.37	< 0.1
	**7 days**	37.9±4.55	< 0.1
**PLA/PEG**	**24 hrs**	38.8±3.55	< 0.2
	**7 days**	39.6±3.27	< 0.2
**PLA/PEG/3CS**	**24 hrs**	37.4±3.89	< 0.2
	**7 days**	38.2±4.64	< 0.1
**PLA/PEG/6CS**	**24 hrs**	37.6±4.33	< 0.1
	**7 days**	39.1±4.46	< 0.2
**PLA/PEG/0.5 R**	**24 hrs**	39.2±4.64	< 0.2
	**7 days**	39.5±3.37	< 0.2
**PLA/PEG/3CS/0.5 R**	**24 hrs**	39.3±5.14	< 0.1
	**7 days**	39.8±5.37	< 0.2
**PLA/PEG/6CS/0.5 R**	**24 hrs**	38.1±4.27	< 0.2
	**7 days**	39.5±5.55	< 0.2

Table 12. The influence of biocomposites administration on the serum complement level and the NBT test.

Groups		**Complement**	**NBT test**
**Control**	**24 hrs**	16.33±1.55	53.73±3.46

	**7 days**	16.48±1.37	53.65±3.55
**PLA/PEG**	**24 hrs**	17.07±0.89	53.85±4.05
	**7 days**	16.63±1.55	53.37±3.64
**PLA/PEG/3CS**	**24 hrs**	16.29±1.72	53.85±3.89
	**7 days**	16.73±1.33	53.45±3.72
**PLA/PEG/6CS**	**24 hrs**	16.85±1.55	53.25±3.64
	**7 days**	16.69±1.33	54.43±3.89
**PLA/PEG/0.5R**	**24 hrs**	17.03±0.83	54.19±4.17
	**7 days**	17.13±1.46	53.49±3.55
**PLA/PEG/3CS/0.5R**	**24 hrs**	17.22±2.04	54.39±4.14
	**7 days**	17.13±1.89	55.46±4.37
**PLA/PEG/6CS/0.5R**	**24 hrs**	17.19±1.37	55.74±4.33
	**7 days**	17.33±1.46	55.48±3.55
